# The effects of a home-based arm ergometry exercise programme on physical fitness, fatigue and activity in polio survivors: protocol for a randomised controlled trial

**DOI:** 10.1186/1471-2377-12-157

**Published:** 2012-12-13

**Authors:** Deirdre Murray, Dara Meldrum, Roisin Moloney, Anna Campion, Frances Horgan, Orla Hardiman

**Affiliations:** 1 Royal College of Surgeons in Ireland, 123 St. Stephen's Green, Dublin 2, Ireland; 2 Physiotherapy Department, Beaumont Hospital, Dublin 9, Ireland; 3 Department of Neuroscience Beaumont Hospital and Trinity College Dublin, Dublin 9, Ireland

**Keywords:** Poliomyelitis, Arm Ergometry, Physical fitness, Fatigue

## Abstract

**Background:**

Many Polio survivors have reduced mobility, pain and fatigue, which make access to conventional forms of aerobic exercise difficult. Inactivity leads to increased risk of health problems, many of which are prevalent among Polio survivors. Aerobic exercise programmes in Polio survivors should utilise stable muscle groups and should be designed to minimise exacerbation of pain and fatigue. A home-based arm ergometry aerobic exercise programme may represent an affordable and accessible exercise modality, incorporating exercise prescription principles in this group.

**Methods/design:**

This is a prospective, single blinded, randomised controlled trial. There are two arms; exercise intervention using arm ergometers and control. Polio survivors meeting eligibility criteria will be recruited and randomly allocated to intervention or control groups. Participants allocated to the intervention group will receive a small arm ergometer and a polar heart rate monitor. They will carry out a home-based moderate intensity (50-70% HRMax) aerobic exercise programme for eight weeks, following instruction by the treating physiotherapist. Assessments will occur at baseline and after eight weeks and will include tests of physical fitness, activity, energy cost of walking, fatigue and quality of life. Clinically feasible assessment tools including the Six Minute Arm Test, the Physical Activity Scale for People with Physical Disabilities questionnaire, the Physiological Cost Index, Fatigue Severity Scale and the SF-36v2 will be utilised.

**Discussion:**

The efficacy of a home-based arm ergometry programme in Polio survivors will be examined. No previous trial has examined such a programme using a wide range of outcome measures pertinent to Polio survivors. This study will provide new information on the impact of arm ergometry on physical fitness, activity, body composition, fatigue, pain, muscle strength, and health related quality of life. Also, the study will provide information, which at present is lacking, on safety of aerobic exercise in Polio, as potential negative outcomes of activity including loss of muscle strength, increased pain and fatigue will be closely monitored.

**Trial registration:**

Clinicaltrials.gov identifier: NCT01271530

## Background

Epidemics of Poliomyelitis in Ireland in the 1940s and 1950s produced an estimated 7,500 Polio survivors, who have since lived with the residual physical deficits associated with their condition [1]. Approximately 50 percent of Polio survivors develop a range of specific symptoms, including new muscle weakness, diagnosed as Postpolio Syndrome [2]. However many more present with reduced mobility, pain and deconditioning, described in the broadest sense as the late effects of Polio. The majority of Polio survivors have some limitation in mobility, which affects their ability to engage in common forms of aerobic exercise, such as walking or cycling. Polio survivors have poor levels of fitness [3], report low activity levels at leisure [4] and have low subjective perception of health [5]. Furthermore, Polio survivors have increased cardiac risk factors such as dislipidemia and increased blood pressure [6].

Recommendations regarding physical activity in older adults issued by the American College of Sports Medicine (ACSM) and the American Heart Association apply to the majority of Polio survivors, as adults over 50 with a clinically significant chronic condition [7]. These recommendations state that older adults should engage in 30 minutes of moderate activity five days per week and emphasise the requirement for an activity plan. A recent survey of 47 Irish Polio survivors found that only 34 percent reported exercising at least three times per week [8]. Fatigue, mobility and pain were the most common reasons for not partaking in regular exercise. In comparison 64% of Irish adults over 45 report moderate to high activity levels, with time identified as the most common barrier [9].

Inactivity leads to an increased risk of health problems including obesity, type 2 diabetes, hypertension, coronary artery disease, respiratory dysfunction and osteoarthritis [10]. Many Irish Polio survivors are overweight [11]. Farbu [12], found that in a cohort of 85 Polio survivors, 32% had a history of cardiovascular disease and 11% pulmonary disease – diseases, which exercise intervention can ameliorate.

Exercise prescription is complicated in Polio survivors, due to concerns that muscle overuse and intensive training could perpetuate further weakness in weak and overused muscles [13]. To date, a small number of trials have found that Polio survivors have the potential to increase their aerobic capacity [14-17]. Treadmill exercise has resulted in an improvement in cardiorespiratory conditioning, with improvements in fatigue and quality of life also reported [16]. A six-month weekly physiotherapy led class, which included five minutes of cardiovascular exercise on cycle ergometers, also resulted in improvements in cardiorespiratory fitness, without ill effect in most participants [17]. However these trials used a range of modes of exercise testing and training, which did not adhere to guidelines for exercise testing and prescription in Polio survivors [14]. A systematic review of exercise in Neuromuscular disease, which included Postpolio syndrome found that the studies examined had insufficient methodological quality [18].

The ACSM [19] recommend that stable muscle groups should be utilised for exercise and that with severe or recent onset weakness should not exercise, while the March of Dimes best practice guidelines [13] recommend not exercising muscle groups where new weakness is being experienced. While well tolerated by some, lower limbs weakness, pain and reduced mobility will commonly limit the ability to exercise comfortably and safely using treadmills or stationary bicycles. Floor or treadmill walking or lower limb cycling may indeed aggravate pain in those with lower limb weakness and altered biomechanics [20]. Training with an ergometer is likely to be an appropriate form of exercise in patients with good, stable upper limb strength. One small trial of ergometry over 16 weeks resulted in a 19% increase in VO2 max in ten Postpolio patients exercising three times per week [15], however the impact on other aspects of health was not examined. In addition arm ergometry has been examined as an appropriate mode of exercise in Polio survivors with subjective rate of perceived exertion significantly related to physiologic markers of exercise intensity [21].

Investigation of responses to aerobic training in patients with neuromuscular disease has been recommended [22]. There is a requirement for well-controlled, prospective trials of aerobic exercise training in Polio survivors, utilising methods of testing and training appropriate to these patients. Ideally those with chronic conditions should be facilitated by health professionals to become as independent as possible in maintaining and improving their physical condition [23]. An appropriate aerobic exercise programme may enable Polio survivors to improve physical fitness, enjoy the health benefits associated with exercise [7] and perhaps reduce fatigue. A convenient and accessible form of exercise is likely to maximise compliance as Polio survivors are advised to exercise when subjective energy levels are good, to avoid exacerbating fatigue [13,14]. A small ergometer, provided for use at home may be an affordable option meeting these requirements.

The aim of this randomised controlled trial is to investigate the effect of an aerobic exercise programme, carried out in the home environment, using arm ergometers, on Polio survivors. We hypothesise that upper limb aerobic training will result in a significant increase in fitness in exercising Polio survivors compared with Polio survivors allocated to a control group. Completing the exercise programme is also predicted to improve activity, energy cost of walking and health related quality of life without increases in pain and fatigue.

## Methods

### Study design

**Figure 1:**
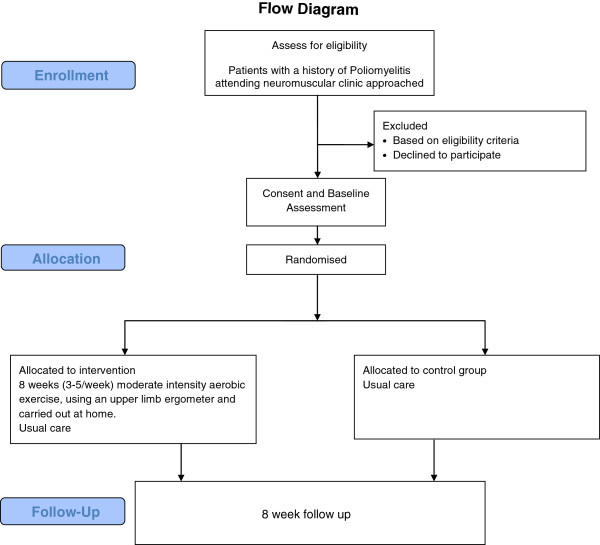
Flow diagram.

This is a prospective, single blinded, randomised controlled trial. There are two arms (i) exercise intervention using arm ergometers and (ii) control. It will not be possible to blind the treating physiotherapist or the patient to the exercise intervention; hence the single (assessor) blinded design. The protocol was approved by the Beaumont Hospital Medical Research ethics committee in November 2009. The trial was designed incorporating recommendations of the CONSORT statement [24]. The flow of participants through the study is represented in Figure 1. All participants will receive usual Physiotherapy care, which does not routinely include arm ergometry. Participants in the control group can choose to cross over to the exercise intervention arm after the eight week control period. Assessments will be completed at baseline and at eight weeks.

### Setting

The study will be based in Beaumont Hospital, Dublin. Assessments will be conducted in the Physiotherapy department and the exercise intervention will be completed in the participants’ homes.

### Participants

Patients will be recruited from medical and physiotherapy post Polio clinics and through advertisement in the national post polio support group publication. Eligibility criteria are shown in Table 1. Information leaflets will be provided, as well as a detailed verbal explanation and questions answered. All potential participants will be screened for suitability by a medical doctor using the PAR-med-X screening tool. A letter will be sent to each recruited patients’ general practitioner, outlining the details of the study and requesting that they contact the researchers should they have any concerns about their patients’ participation. Prior to baseline assessment written informed consent will be obtained. Participants will be free to withdraw at any time.

**Table 1:** Eligibility criteria

**Inclusion criteria**	
1	A history of Poliomyelitis affecting at least one lower limb, confirmed by the Neurologist
2	Capable of walking for 6 minutes, with or without an aid/appliance
3	Good upper limb strength, confirmed objectively using maximum voluntary isometric contraction, 7 out of 10 tested upper limb movements must lie above the 5th percentile of the normal range
4	Completion of the PAR-Med-X assessment and cleared by a medical practitioner as safe for exercise
5	Males/Females aged 18-75 years
**Exclusion Criteria**	
1	Unstable cardiac or respiratory conditions, including oxygen dependence
2	Uncontrolled hypertension
3	Significant upper limb pain. Greater than 4/10 on a Visual Analogue Scale or more than 3 specific sites of pain in the upper limbs, neck or upper back
4	Severe fatigue (greater than five on the Fatigue Severity Scale)
5	Recent onset of upper limb weakness or severe upper limb weakness
6	Steroid use in last 3 months
7	Pregnancy

### Sample size

The sample size for the study was determined based on a hypothesised change in heart rate of eight beats per minute, during the six minute arm test. Change in fatigue was also examined. The change in heart rate was based on reported changes in previous exercise interventions and considered clinically significant [17,25]. For each calculation power was set at 80%, alpha at 5% and drop-out rate at 15%. The formula N= 2 [(standard deviation)^2^/(difference)^2^ x 7.9 was used. We aim to recruit 120 participants (60 per group). Table 2 outlines the number of participants required based on the six minute arm test and the fatigue severity scale.

**Table 2:** Sample size calculation

**Outcome measure**	**Clinically significant difference**	**Standard deviation**	**Number of participants required per group**
6MAT (Heart Rate)	8 b/m	14.5	60
FSS	1 point	1.1	48

### Randomisation

The randomisation sequence will be created prior to study commencement using http://www.randomization.com. A 1:1 allocation in block sizes of 10 and stratified by gender will be used. Group allocation will be concealed from the blinded assessor using sequentially numbered sealed opaque envelopes, colour coded by gender. Envelopes will be opened with the participant by the treating physiotherapist, after baseline assessments have been completed. Gender stratification is necessary to avoid weighting of sexes to any particular group, which may skew physical fitness data.

### Intervention

Participants randomised to the intervention group will exercise at home using arm ergometers for eight weeks. The treating physiotherapist will provide an individualised exercise programme during a home visit. Participants will receive a static cycle, which placed on a table is used as an arm ergometer, a polar heart rate monitor and an exercise log/instruction booklet.

Participants will begin exercising for at least ten minutes per day three days per week. If an individual has difficulty exercising for 10 minutes continuously, the 10 minute session may be broken into two to three minute bursts of exercise, with one minute rests [15,26]. Participants will aim to increase exercise durations to 30 minutes per day three to five days per week. Participants will be educated regarding the target of ACSM recommendations of a cumulative 150 minutes of moderate intensity exercise per week [7].

Participants will exercise at a moderate intensity [7,27]. Maximum heart rate (MaxHR=220-age) will be used to prescribe a target exercising heart rate of 50-70% HRMax in addition to a subjective level of 13–17 on the BORG rate of perceived exertion (RPE). This exercise prescription meets recommendations for exercise in Polio survivors and is similar to those previously conducted and tolerated in this group [16,19]. Prescription using HRMax has been recommended for use in this group as difficulties have been reported in achieving target heart rate using Karvonen’s formula, due to high resting heart rates [15].

Exercise intensity will be modified by changing pedal rate or resistance and monitored using the polar heart rate monitors. The treating physiotherapist will educate participants on how to alter exercise intensity based on exercising heart rate. Exercise time, heart rate and RPE will be recorded by the participant in the exercise log for each exercise session. Participants will pedal with zero resistance for 2 minutes prior to the exercise session, to warm up and will be provided with upper limb range of movement exercises and stretches as cool down exercises.

Participants will monitor fatigue and pain on 0–10 Visual Analogue Scales. If there is an increase of more than 2 points in upper limb, neck or back pain, or subjective fatigue, the participant will be advised to reduce exercise duration and/or intensity. If this does not result in an improvement, the participant will be advised to contact the treating physiotherapist by phone. This physiotherapist will then make a decision on how to proceed and the participant will be managed appropriately and withdrawn from the study if necessary. All adverse events will be recorded and managed according to a detailed policy. Participants randomised to the control group will be advised to continue with normal activities and will receive physiotherapy care as appropriate.

### Outcomes

Assessments pre- and post the intervention or control period will be conducted by the same blinded assessor (physiotherapist). Demographic data (age, gender, height, weight,) including information specific to history of Polio and mobility will be gathered at the baseline assessment. A combination of self-report questionnaires and objective measurements will be used at baseline and follow-up. An exit questionnaire, requesting feedback on participant’s experiences will be provided at the end of the participation period.

The primary outcome measure will be a change in physical fitness measured using the Six-Minute Arm Test (6-MAT) [29]. This is a submaximal exercise test conducted using upper limb ergometry. Heart rate and rate of perceived exertion using the BORG 6–20 scale (RPE) are recorded during six minutes of arm cycling at a predetermined power output, based on physical ability. This test was developed for patients with spinal cord injury and has acceptable test-retest reliability and validity. Power output levels were adapted from those defined for spinal cord injury patients to suit the profile of Polio survivors.

The secondary outcome measures will be changes in

(i) Self reported physical activity, assessed using The Physical Activity Scale for People with Physical Disabilities questionnaire [30,31]. Validity of this questionnaire has been examined and found acceptable in a group of patients with disability, including Polio survivors.

(ii) Body composition, assessed using Body Mass Index and waist to hip ratio as described by ACSM [32].

(iii) Energy cost of walking, using the Physiological Cost Index (PCI). The PCI was developed by MacGregor and combines assessment of heart rate and walking speed [33]. There are varying reports of validity and reliability in different patient groups, for example it is valid and reproducible (ICC=0.89) in children with cerebral palsy, although less sensitive than direct measurement of oxygen cost [34]. The PCI is a clinically accessible proxy measure of energy cost, not requiring expensive equipment. It was previously successfully used in a similar group of Polio survivors [35].

(iv) Fatigue measured using the Fatigue Severity Scale (FSS) [36,37]. The FSS is reported as being applicable to Polio survivors with a better ability to distinguish those with disabling fatigue from those without [37]. Reproducibility of the FSS in Polio survivors is good (ICC 0.83) [38].

(v) Quality of Life using the SF-36v2 [39,40] . The SF-36 is a measure of health status and quality of life, and has been used in several studies in Polio survivors [39-41].

(vi) Pain, assessed using the Short Form McGill Pain questionnaire version 2 [42], body diagrams to document pain location and visual analogue scales. Visual analogue scales and body diagrams have been used in several studies of Polio survivors [20,43-45].

(vii) Upper limb strength (maximum voluntary isometric contraction, KGs), measured using fixed dynamometry using the Quantitative Muscle Analysis (QMA) system [46]. Shoulder abduction, adduction, elbow flexion and extension and hand grip will be measured using strictly standardised methods previously described [46-48]. Each test is completed if the participant has adequate power to generate a resistance against the load cell or dynamometer. If not a score of zero is given.

(viii) Hand grip motor fatigue**,** measured using QMA [48].

### Statistical analysis

Data will be coded and collated in a Microsoft Excel® spreadsheet and Stata 12 will be used for statistical analysis. All analyses will be conducted on an intention-to-treat principle [49]. Missing data will be managed using the last observation carried forward method. Per-protocol analyses will be performed excluding participants with major deviations from the treatment protocol (defined as less than 50% compliance with treatment). Demographic characteristics and baseline data will be summarised using descriptive statistics and baseline comparability of the groups will be examined. The distribution of the data will be assessed and if appropriate, parametric methods will be used in analysis. Two sample t-tests will be used to compare groups and paired t-tests will be used for within group comparisons. The non-parametric equivalent will be used where data are not normally distributed. A significance level of p<0.05 will be set. The primary analysis will examine the difference between intervention and control groups. A secondary analysis will compare pre and post intervention scores and will include control participants who crossed over to the intervention group.

## Discussion

This protocol has detailed the process by which the efficacy of a home-based arm ergometry programme in Polio survivors will be examined. No previous trial has examined such a programme using a wide range of outcome measures pertinent to Polio survivors. This study will provide new information on the potential for arm ergometry to form part of a treatment regimen for Polio survivors and will examine the impact on physical fitness, activity, body composition, fatigue, pain, muscle strength, and health related quality of life. Also, the study will provide information, which at present is lacking, on safety of aerobic exercise in Polio survivors, as potential negative outcomes of activity including loss of muscle strength, increased pain and fatigue will be closely monitored. The use of outcome measures that are clinically accessible will add internal validity to the study. The home-based exercise programme, utilising affordable items of equipment, has the potential to facilitate aerobic exercise in Polio survivors, allowing this patient group to take advantage of the benefits of exercise.

## Abbreviations

ACSM: American College of Sports Medicine; MaxHR: Maximum Heart Rate; RPE: Rate Of Perceived Exertion; 6-MAT: Six Minute Arm Test; PCI: Physiological Cost Index; FSS: Fatigue Severity Scale; QMA: Quantitative Muscle Analysis.

## Competing interests

The authors declare no competing interests.

## Authors' contributions

D Murray, D Meldrum and O Hardiman conceived and designed the study. D Meldrum drew up and performed the randomisation schedule. D Meldrum, F Horgan and O Hardiman evaluated and revised the protocol for intellectual content. D Murray formulated the interventions for the study and is the treating Physiotherapist. R Moloney and A Campion are the blinded assessors. All authors read and approved the final manuscript.

## Pre-publication history

The pre-publication history for this paper can be accessed here:

http://www.biomedcentral.com/1471-2377/12/157/prepub
